# Valedectory Address to the Graduates of Rush Medical College, Session 1855–6

**Published:** 1856-05

**Authors:** W. B. Herrick

**Affiliations:** Professor of Physiology and Pathology


### THE NORTH-WESTERN

### MEDICAL AND SURGICAL JOURNAL.

(NEW SERIES.)

VOL. V. MAY, 1856. NO. 5.

### ORIGINAL COMMUNICATIONS.

ARTICLE I.—*Valedictory Address to the Graduates of Rush Medical College, Session of* 1855–6. By W. B. HERRICK, M.D., Professor of Physiology and Pathology. [Published by request of the Graduating Class.]

In compliance with the request of our President, and in behalf of my colleagues, I rise to perform a duty which we, as teachers, owe to ourselves, the public, and especially to you, gentlemen, who have this evening assumed the high responsibilities of a profession, the importance of which experienced practitioners and teachers alone can appreciate.

Believing that, in justice to ourselves and the noble science of our adoption, we should, on all proper occasions, advocate its claims, especially upon such as are, as its representatives, about to become responsible for the degree of honor and respect paid to it by others; I propose, for the purpose of showing, by this portion of its history, what are its claims to both public and private consideration, at this time to give to my hearers, and especially to those of the graduating class, a brief historical sketch of the origin of the science of medicine; for all must admit, that in proportion as any profession, art or science has an influence upon the condition and progress of man, as indicated by past history, present considerations or future anticipations, it merits honor and respect.

The Temple of Medicine, viewed by the light of such historical facts as are now before us, seems to have been progressing towards completion during several distinct periods; separated by intervals during which the laborers, disheartened for want of the materials now abundantly supplied by the physical sciences, especially anatomy and chemistry, abandoned for a time the noble work, and suffered its unfinished and neglected walls to crumble and decay under the destroying influence of time and neglect.

The first of these—extending from as far back as tradition and history can carry us, to the dispersion of the Pythagorian society 500 years before Christ—is remarkable for being that in which all knowledge was communicated from the teacher shrouded in mystery, and received by the learner with blind credulity.

We learn from sacred history, that for a period immediately following the creation of man, and at intervals for a long time after, our Heavenly Father, as if mindful of the helpless condition of his children in their yet imperfect and infantile state, communicated to them, either directly or by inspiration, the knowledge requisite for the preservation of health and prevention of disease.

"The writings of Moses," says a distinguished medical historian, "constitute a precious monument of the history of medicine," for they embrace hygienic rules of the highest sagacity.

Moses, it would seem, communicated these hygienic laws to the priests, the Levites, and they to the people. This was the practice among the Jews till up to a period 300 years before Christ, when physicians, having a true and independent position as students of nature and cultivators of science, received from one of the inspired writers of the Bible itself the following high commendation:—

"Honor the physician, for he is indispensable, for the Most High has created him."

"For all medicine is a gift from God, and the physician shall receive homage from the king."

"The science of medicine shall elevate the physician to honor, and he shall be praised before the great."

"The Most High has created the medicines out of the earth; and he that is wise will not abhor them."

Ere long, however, man, preferring to be guided by his own wayward inclinations rather than to submit longer to the Divine laws, had wandered from that stream, the source of which was an ever-living inexhaustible fountain of knowledge and truth, at a time when the people of all nations, whether Jews or Pagans, were seeking to quench their natural thirst for knowledge from those impure fountains furnished by the priests; who, in Jewish synagogues and Pagan temples, assumed to be the only true physicians for both body and soul, and to possess all knowledge of the past, present, and future, as is abundantly shown by the historical and traditional accounts of ancient medicine, as it then existed throughout the world.

The antiquity of Egyptian Medicine is shown from the fact, that 1700 years before Christ, Egypt possessed men who practiced the art of medicine, for we read in the Bible, that when Jacob died, "Joseph commanded his servants, the physicians, to embalm him; and the physicians embalmed Israel, and forty days were fulfilled for him; for so are fulfilled the days of those that are embalmed."

Thus it is seen, that embalming was in the hands of Egyptian physicians, yet so averse were the people to anatomical investigations, that they stoned those whose business it was to make the opening through which the material was introduced, and thus compelled them to flee for safety.

"Thoth, whom the Greeks named Hermes, and the Latins Mercurius, passed among the Egyptians as the inventor of all science and art, and was said to be the author of thousands of volumes on various scientific subjects; of which those upon medicine helped constitute a volume called the *Sacred Book*, written in a language known to the priests alone, and containing rules, to deviate from which was death."

In India, the Brahmins were both priests and physicians.

They alone were permitted to know Sanscrit, the language of the learned, in which all medical books were written.

During their professional visits to patients, they examined the pulse, observed the countenance, looked at the stars, watched the flight of birds, &c., but never noted the symptoms. They were especially attentive to cleanliness and rules of hygiene; but assumed, with all the presumption of a Mormon prophet, that their knowledge was directly from the gods.

All that is known of the condition of medical science among the Greeks, previous to the Trojan war, is traditionary, and so blended with the mythology of that period that it is impossible to distinguish, through the shadowy veil which poetic and imaginative historians have drawn between the present and past, who of the numerous gods and deified men of that mystic age were the real fathers of Medicine.

According to their mythological traditions, *Melamphus* was the first Greek physician "who immortalized himself by extraordinary cures, and to whom, from gratitude, altars were erected." He lived in the time of Proteus, king of Argos, nearly 200 years before the Trojan war. He is said to have cured Iphiclus of a species of debility by giving rust of iron; and the daughters of Proteus of hysteria, with milk in which white hellebore had been infused. (Not bad practice that, for a physician who lived more than 3000 years ago.)

Chiron, another of the antique physicians, was less illustrious than his contemporaries as a practitioner; but more noted than any as a teacher. He held his school in a grotto in Thessaly, and, if the tradition be correct, we modern professors might well envy the high and honorable position of old Prof. Chiron, who could number among his pupils many of the heroes concerned in the capture of the Golden Fleece, and in the Trojan war—such as, the mighty Hercules, the subtle Ulysses, the fiery Diomedes, the prolix Nestor, the pious Æneas, and the invincible Achilles. But, of all his disciples, the most noted was Esculapius, whose devotion during his eventful life to the science of medicine, then in its infancy, caused him to be deified by the Greeks as the son of Apollo; believing, as they did at that period, that any new development in the arts and sciences could have no other than a divine origin. Hence, Esculapius was regarded with nearly universal veneration. His worship, which passed from the Greeks to the Romans, extended into all countries penetrated by the arms of these two nations. Throughout the then civilized world numerous temples were erected to his honor, in which medical teachers, as priests, were constantly employed in perpetuating his doctrines and cherishing the yet young and undeveloped son, committed to their care by the Father of Medicine.

In regard to the treatment of diseases by this ancient physician, we possess no information entitled to much credit. The poet Pinder, who lived 7 or 800 years after, says of Esculapius: "He cured the ulcers, wounds, and fevers of all who applied to him, by enchantment, calming potions, incisions, and by external applications." It is said, he brought to life, Hippolytus, son of Theseus; a Capaneous, a Lycurgus, an Eryphile, and many others.

Pluto, god of the infernal regions, alarmed to see the number of new arrivals in his gloomy kingdom diminishing day by day, complained to Jupiter, who destroyed the audacious healer. On this account, says a wit, modern sons of Esculapius abstain from performing prodigies.

Other nations, both of the old and new world, have traditions showing that the practice of medicine began with the formation of society, and that it has, everywhere and in all time, been held in high veneration.

"In Gaul, and in the Brittanic Isles, the Druids were at the same time priests, legislators, and physicians."

Montezuma, emperor of Mexico, possessed gardens where great numbers of plants were cultivated, the properties of which were well known to the physicians of the country.

Thus we have seen that the first notions of medicine go back to the earliest infancy of society in all the countries of the world, and if there exists any nation which, at any epoch in its history, was without physicians, there is not one in which we do not find some vestige of medicine.

The following remarks of the wise Socrates, upon the diseases consequent upon a change from the simple and primitive habits of the people of the age of Hippocrates, to those of refinement and luxury, such as were indulged in many years after by the people of an *ancient republic*, merit from us, members of this great confederacy, equal consideration: "Is it not a shameful thing," says he, "to be compelled to call upon a physician, not for the cure of wounds or diseases of the seasons, but for such as are produced by indulgent life, which fills us with humors and unhealthy vapors like swamps, thus obliging the worthy sons of Esculapius to invent such new names as catarrhs, fluxions, etc.?"

A certain portion of this period of the foundation of medicine, extending from the time of the Trojan war to that of Pythagoras, was characterized as that during which the worship of Esculapius, as the god of medicine, was spread throughout Greece, Asia, Africa, and Italy.

Among the temples which were consecrated to him, those of Epidaurus, Cos, and Cyrene are particularly remarkable.

The priests attached to his worship were named Esculapiadæ, a word which signifies descendants of Esculapius.

They formed a particular cast, governed by sacred laws, like the priests of Egypt, one of which is as follows:—

"It is not permitted to reveal sacred things, except to the elect; and strangers must be admitted to this knowledge only after having submitted to the tests of initiation."

"The temples of the god of medicine," says a medical historian, "were generally very salubriously situated; sometimes on the summit of a hill, or the declivity of a mountain; sometimes on the shore, somewhat distant from the sea, and near a thermal spring or fountain of living water. Groves of trees refreshed the sight of the sick, and afforded to them cool and solitary retreats in their beautiful and spacious avenues.

The people came from all quarters on pilgrimages to those places sacred to the god of medicine. The sick and convalescent found there both agreeable and healthful diversions. The wholesome regimen to which they were subjected, the pure and temperate air they breathed, the faith and hope by which some were animated, the miraculous cures which were testified to—*all* united to affect their minds agreeably, and to exercise a happy influence on their constitutions.

Knowing the great influence of the imagination, these priest-doctors employed every means to control the minds of the patients, they not being permitted to interrogate the oracle until they were purified by washing, abstinence, fastings, prayers, and sacrifices.

"The patients who recovered went to their homes, blessing the divine author of their recovery, and leaving behind them testimonials of their gratitude.

Those who received no benefit, believing that their offerings were rejected because insufficient, redoubled their zeal and liberality; so that bad as well as good results added equally to the glory of the god and the profit of his ministers.

We have been thus explicit in describing the deceptive cunning of these ancient priest-doctors, in order to show the remarkable correspondence of the course pursued by them with that adopted by numerous modern illegitimate sons of Esculapius, who, in numerous cunningly selected salubrious and attractive localities throughout our country, have erected Homœopathic, Hydropathic, and Eclectic temples, in honor of the first propagators of their false and base assumptions; where, in return for liberal sacrifices of both time and money, fashionable, ignorant, and credulous votaries are made to believe, that health is secured and life prolonged by their inefficient and often injurious practice, rather than from the pure air of heaven, the life-giving fruits from the generous earth, the crystal streams from the ever-living fountains, and the beautiful mind-soothing prospects presented everywhere, above, beneath, and around us; which are the only true remedial agents for diseases of both mind and body, prescribed for and freely dispensed to his ungrateful children by the great physician, our Heavenly Father.

To return again to our medical history, it is evident from what has been stated, that, for a space of about 700 years, the science of medicine remained nearly dormant, wrapped up in a mysterious garb, in the hands of the Esculapiadæ or priests, whose crude doctrines were propagated and consultations held in the name of divinity in temples and caves dedicated to the to the god of medicine, Esculapius.

Finally, towards the close of the period under consideration, the foundation of the medical edifice, which had thus far been laid for the most part with mis-shapen material, taken at hazard and without taste or method, begins to rise, grand and majestic, gradually harmonizing in all its parts; for reason and genius have united to improve what accident and instinct had previously suggested.

The great philosopher *Pythagoras,* with his followers the so called Pythagorians, now appeared upon life's busy stage, and the scenes were once more shifted for the performance of another act in the great historical drama of medical progress. To the followers of Pythagoras belongs the honor of having been the first among the sons of Esculapius to reject with scorn the aid and support of an hypocritical priesthood.

Up to this time, the temples of Esculapius had been both schools for the student and dispensaries for the sick; but now the era of mysticism in medicine had passed away, and the reign of intelligence had began.

The gymnasiums no longer, as formerly, dedicated to physical exercise alone, became schools for the development of mind and the cultivation of medicine and the kindred sciences.

The next most distinguished laborer in the construction of the edifice, to which this new impulse had been given, was *Hippocrates,* born at Cos 469 years before Christ, of a family in which it was said the practice of medicine was hereditary, seven of them having been at different periods practitioners or teachers.

He was educated at Cos, one of the most noted Esculapian schools, after which he traveled into the various countries in which the science of medicine had been cultivated.

After his return to his native country, being rich in the materials he had collected, he published those immortal works that astonished the world, and made the physical science of man one of the most important branches of natural philosophy.

During his time, and for many years after, theoretical dogmatism exercised a tyrannical sway over the minds of physicians, compelling them to deviate from the more certain paths of observation and experience, and follow the then numerous false beacon-lights set up by Hippocrates and other learned but imaginative writers, among whom may be mentioned as most prominent, Plato and Aristotle, whose doctrines have exerted a great influence upon the march of mind, and especially upon the progress of medicine.

An idea of the attractive beauty of the then extensively popular Platonic philosophy, may be drawn from the following quotations from the writings of this brilliant and imaginative philosopher.

"Is anything more rational than to think by the thoughts alone, disengaged from all foreign or sensible agency; to apply at once the pure essence of thought in itself, without the ministry of the eyes and ears; without, in short, any intervention of the body, whose slightest influence only troubles the soul and prevents its finding wisdom and truth."

"I comprehend no longer all the other learned causes that are offered us, but if any one comes to tell me what it is that makes a thing beautiful, or gives it loveliness of colors or forms, or other similar things; I leave aside all these reasons which only perplex me, and I assume myself without plan or art, and probably too simply, that nothing gives beauty but the presence or communication of the original beauty. I affirm, nothing, except that all things beautiful are beautiful by the presence of beauty."

It was at a time when Pythagoras recommended, in secret, to his disciples, and when Plato taught publicly, with all the prestige of his imagination and eloquence — that contemplative philosophy, which assumed that the senses transmit false, doubtful, or illusory impressions, and that the mind, to attain truth and certainty, must isolate itself as much as possible from the body: that a pupil of Plato, (afterwards tutor to Alexander the Great, and finally the greatest of ancient philosophers,) *Aristotle*, stepped forth into the arena of science, and boldly announced, for the first time, that famous axiom, "All ideas come from the senses," and thus introduced into the philosophic world a new principle in manifest contradiction to the received dogmas of Pythagoras and Plato.

It appears, says he, that the "remembrance of things often repeated, creates experience: and experience, that is to say, every general notion which becomes fixed in the soul relative to the common properties of things, is the principle of science and art.

"To possess the knowledge that a remedy has been useful in the hands of Socrates and several others, is experience; but to know that such a remedy is good for all individuals of the same species, attacked by a given affection, is what constitues art."

The above quotations are sufficient to show, that there have been but few if any minds since his time, equal in capacity to that of this ancient philosopher; yet, for want of anatomical and chemical knowledge, his physiological conclusions, viewed by these great lights in medicine now burning so brilliantly, seem most absurd and unworthy of such an origin.

"The nerves," says he, "proceed from the heart, that viscus containing nerves in its structure, in its largest cavities; and what is termed the aorta is a nervous vein, whose extremities are nothing else than nerves.

"The principal nerves, are those of the ham, on which depends the power of leaping; and another double nerve called the tendon, and then the extensor and the nerve of the shoulder, which contribute to the strength of the body."

Such opinions as these from such a source seem most absurd, especially when we know, as we do, from history, that Aristotle had dissected a considerable number of animals of every species, and compares, with a sagacity astonishing for his era, the organic apparatus by means of which each of them lives and fulfills its various functions.

His position among his contemporaries may be inferred from the following quotation from an ancient satirist, who makes Mercury say: "Behold a man, who can tell you in a moment the duration of the life of a fly; to what depth the rays of sun penetrate the sea; and what is the nature of the soul of an oyster."

The last and most important portion of this period of the foundation of medicine, began about the termination of the wide-spread conquests of Alexander the Great; who, after subduing and bringing under subjection nearly all the physical force of the then known world, was equally ambitious to control and direct the march of mind, by making the capital of his vast empire the great centre for philosophical discussions and the development of the arts and sciences.

He, therefore, caused to be collected from the various parts of his wide domain all the manuscripts upon scientific subjects, and especially those upon medicine, with which was formed that wonderful collection of half a million of volumes, the Alexandrian library.

It is also said of this great conqueror, that, in the midst of the turmoil and excitement of war, he kept up a scientific correspondence with Aristotle, his former teacher; and not only furnished the necessary funds for that purpose, but occupied himself in the collection of animals and plants, and every sort of rare object, from the depths of Asia, from which was formed the first great museum of natural history.

This, with the library, were placed at the disposal of literary and scientific men of all nations, who were invited to this great centre of literature and science, furnished with rooms in or near the library and museum, and supported by a fund set apart for that purpose.

Some were charged with the collection and arrangement of manuscripts and specimens in natural history; others, with scientific and philosophic investigations; whilst all were required to meet at the museum on certain days, at what were called literary contests or feasts, to deliver lectures and discuss various subjects; after which, those who succeeded best received public eulogies and rewards proportionate to their merit.

Galen, who was one of the most noted among those who devoted themselves to investigations, with special reference to the advancement of medical science, is the only author of that period whose writings on anatomy, physiology, and other branches of medicine, have come down to us of the present day.

It would seem, from what we can now infer from the writings of this, the most distinguished of the numerous talented physicians of the Alexandrian school, that the success which then attended the cultivation of medicine, was due to the authorization of dissections of the human body, never before practiced without peril, but now not only permitted but encouraged even by sovereigns above the prejudices of the age.

Stripped of its mystic veil, the temple of medicine now takes a sudden progressive step.

All the materials for its construction, excepting that great corner stone, the discovery of the circulation of the blood, had been provided, and it only remained for William Harvey, one of our Anglo-Saxon progenitors who lived near the beginning of the 17th century, to bring forth from beneath the rubbish of demolished false opinions of that day, this much required constituent of a noble structure.

Thus it seems, that instinct, accident, and observation furnished the first materials for the foundation of our medical edifice, and that reason came next to polish, cull, and arrange these materials, and direct investigation and experience in laying the corner stone and the construction of its walls.

From this short review of medical progress during the age of its foundation, it will be seen that then, as now, two modes of acquiring knowledge were recognized:—

*First.* The logical or rational, which consists in establishing as a basis certain propositions or abstract principles, (*axioms,*) whence is deduced by reason the solution of particular causes or facts.

*Second.* The empirical or experimental, which consists in observing a great number of causes or facts, which being compared, classified, and made to harmonize, express certain propositions or abstract principles—*axioms.*

The first method, as it is seen, proceeds from generals to particulars, from the abstract to the concrete, from the axiom to the phenomenon.

The second, on the contrary, advances from particulars to generals, from the concrete to the abstract, from the phenomenon to the *axiom.*

Far from being opposed to each other, as was contended by ancient medical philosophers, these two methods fortify and enlighten each other; and the truth never takes hold of us in a more irresistible manner than when it is obtained by both routes at once, as is abundantly proven by the wonderful progress of the temple of medicine towards completion since this doctrine began to be recognized, as it now is, by our best medical writers and teachers.

An occasion like the present is not one admitting of a more extended view of historic medicine, we will now, therefore, after one other reflection, close our remarks upon this, to me, interesting subject, to do justice to which would require more time and study than most of us can command.

The facts upon this subject, as far as presented, show most conclusively that *Medicine*, like all the sciences based upon facts derived from the study of nature's undeviating laws and the careful observance of natural phenomena, has kept pace with the march of mind from its primeval state, when, like a child guided by the hand of parental love, its implicit confidence in the stability of truth had never been shaken to the present time, when far advanced on its journey in pursuit of knowledge, it has learned to watch for false beacon lights, deviating paths, and hidden pit-falls, which have so often led astray and retarded the progress of the ambitious and truth-seeking student, in his toilsome journey up the steep and rugged path to that Temple of ours, the pure light shining upon which is even now seen in the distance, indicating a nearer approach to that happy period when the rays of truth, unobstructed by false views and theories, will shine upon it with all their primitive brightness.

